# Improvement of Thermal Stability of BCG Vaccine

**DOI:** 10.18869/acadpub.ibj.21.6.406

**Published:** 2017-11

**Authors:** Fatemeh Jahanbakhsh Sefidi, Homan Kaghazian, Gholam Ali Moradli, Seyed Mehdi Hassanzadeh

**Affiliations:** 1 Department of Microbiology, College of Basic Science, Islamic Azad University, Saveh, Iran; 2 Research and Production Complex, Pasteur Institute of Iran, Tehran, Iran

**Keywords:** BCG vaccine, Quality control, Stability

## Abstract

**Background:**

Thermal stability (TS) is a part of the BCG vaccine characterization by which the consistency of process in BCG vaccine production could be confirmed. To enhance the TS of the vaccine, some prevalent stabilizers in different concentrations were added to the final formulation of BCG bulk prior to Freeze-drying process. We found a formulation more effective than the current stabilizer for retaining the higher viability of lyophilized BCG vaccine produced by Pasteur Institute of Iran.

**Methods:**

In the design of experiments using Taguchi method, lactose, trehalose, glucose, dextran, and monosodium glutamate were added to the final formulation of BCG bulk prior to freeze-drying process. Viability of the samples was determined by counting the colony forming unit.

**Results:**

Maximum signal-to-noise ratio equal to maximum TS and viability was obtained by adding lactose, dextran, and glutamate in defined concentrations.

**Conclusion:**

Adding the stabilizers had a significant impact on TS of BCG vaccine to meet the quality requirements.

## INTRODUCTION

*Mycobacterium bovis* BCG (Bacille Calmette-Guérin) is the only approved vaccine against tuberculosis (TB) from the beginning of 1921 up to now[1-4]. Most freeze-dried BCG vaccines are stable at 2-8°C for at least two years; however, the stability varies at room temperature, and after storage for several months, a loss of viability of approximately 30% can be expected[5,6].

Thermal stability (TS) has been considered as one of the most important quality parameters of a freeze-dried BCG vaccine for each final lot release[7,8]. TS test is a part of the characterization and consistency demonstration of the BCG vaccine production and also could be important for transportation and storage of the vaccine in a tropical area[9]. Viability (colony-forming units [CFUs]) of the vaccine incubated at 37°C for 28 days should not be less than 20% of the similar vaccine that stored at 2-8°C for 28 days[6,9]. TS of the vaccine depends on the variety of BCG strain, cultivation method, production technique, nature of stabilizer, moisture content, primary packaging material and freeze-drying process[4,10]. Therefore, TS of BCG vaccine has been investigated in many studies[4,5,7,9,11-13]. All available BCG strains with different TS are derived from the one produced by Calmette [6]. It is not clear if TS is strain-dependent or dependent on the lyophilization and stabilization techniques of the manufacturers, though it is probably the latter[6]. Effect of stabilizers including some carbohydrates and amino acids in the formulation of some BCG vaccines have already been investigated, and some of them have been approved by their regulatory authorities[7,9,12-16].

The objective of this study was to improve the TS of the freeze-dried BCG vaccine producing at Pasteur Institute of Iran by adding some stabilizers, including glucose, lactose, trehalose, dextran, and L-glutamate. Taguchi method (MINITAB version 14) was used to design the experiments for optimization of concentration of the stabilizers.

## MATERIALS AND METHODS

### Materials

Mycobacterium bovis BCG 1173P2 was obtained from Pasteur Institute of Paris (France). All chemicals used in the culture medium or in vaccine preparation were purchased from Merck Co. (Germany).

### Final bulk of vaccine production

A vial of freeze-dried working seed lot of BCG vaccine was reconstituted and inoculated on the surface of Sauton medium following incubation at 37°C for 20 days. The proliferated bacilli in form of pellicle were passaged two times more on the surface of Sauton medium. Subsequently, the Bacterial pellicle was collected and passed through the filtration system to obtain a semi-dry biomass. Biomass was weighted and suspended by adding 1.5% L-glutamate solution to obtain the final bulk of vaccine (2 mg/ml) and then kept in 4-8ºC.

### Formulation of final bulk with various concentrations of stabilizers

According to Taguchi DOE (Minitab 17), the proper orthogonal array (L16) was selected to examine 16 formulations with 4 stabilizers, each one at four levels of concentration (0, 0.5, 1, and 2%) in order to achieve an optimized final formulation of BCG vaccine prior to freeze drying. To determine the effect of each variable factor (stabilizers) on the output (CFU), the higher signal-to-noise (S/N) ratio was the best, and it was calculated for each of the 16 experiments.

### Preparation of BCG vaccine

Vials filled with 0.5 ml of each formulation of final bulks were freeze-drayed according to the verified procedure and program.

### Thermal stability of BCG vaccine

The samples of BCG vaccines with different final formulations were incubated at 5ºC for 28 days and at 37°C prior to reconstitution and viability test. CFUs, as the viability of the samples, were then determined.

### Measurement of bacterial viability

The samples of freeze-dried vaccine were reconstituted with Sauton medium to obtain final concentration of 1 mg/ml, and the suspension was diluted more to obtain dilutions rates of 1.8×10^-4^. Diluted solutions were inoculated on the surface of the Lowenstein–Jensen medium in a tube and incubated at 37°C for 21-35 days. CFU were counted in weeks 3, 4, and 5 after incubation.

## RESULTS

Four levels were assigned to five factors according to the L16 Taguchi array to select the most effective concentrations of the stabilizers for optimization of TS in 16 separate experiments. Results from the achieved CFU showed that the maximum S/N ratio, viability, and heat stability were obtained in the experiment No.10, in which the concentrations were at the levels of 3 for sodium L-glutamate, 2 for glucose, 4 for dextran, 3 for lactose, and 1 for trehalose. These levels were equal to 1, 0.5, 2, and 0 (%) of each corresponding compound (Table 1).

**Table 1:** Levels assigned to the factors studied according to the L16 Taguchi array and concentration of stabilizers & response values for the CFUs

Trial	Factors and levels					CFU×10^6/mg			Thermal stability	S/N	STD	Mean	CV
	
	N	G	D	L	T	1	2	3					
1	1	1	1	1	1	2	2.1	2.2	4	6.4	0.1	2.1	0.05
2	1	2	2	2	2	4	3.8	3.7	7	12	0.2	3.8	0.04
3	1	3	3	3	3	8.1	7.9	8.3	17	18	0.2	8.1	0.02
4	1	4	4	4	4	8.9	8.8	9.3	17	19	0.3	9	0.03
5	2	1	2	3	4	10	9.5	9.7	19	20	0.3	9.7	0.03
6	2	2	1	4	3	11	12	11	18	21	0.6	11.2	0.05
7	2	3	4	1	2	8.1	7.9	8.3	16	18	0.2	8.1	0.02
8	2	4	3	2	1	8.4	8	7.8	15	18	0.3	8	0.04
9	3	1	3	4	2	12	12	12	21	22	0.3	12	0.02
10	3	2	4	3	1	16	16	17	26	24	0.4	16.2	0.02
11	3	3	1	2	4	12	12	12	21	22	0.2	12	0.02
12	3	4	2	1	3	11	11	12	19	21	0.5	11.2	0.04
13	4	1	4	2	3	7.8	8.3	8.7	15	18	0.5	8.2	0.05
14	4	2	3	1	4	6	6.3	5.5	11	15	0.4	5.9	0.07
15	4	3	2	4	1	5.5	5.3	5.9	9	15	0.3	5.5	0.05
16	4	4	1	3	2	5	4.9	5.3	10	14	0.2	5	0.04

N, sodium L-glutamate; G, glucose (+D); D, dextran 40; L, lactose (+D); T, trehalose; S/N, signal-to-noise; STD, standard deviation; CV, coefficient variation

Standard deviation in the triplicate experiments seems to be higher than most of the bacteriological quantitation tests because of aggregation form of BCG bacilli that make more deviation in viable particles count and accordingly different CFU on the culture medium.

As shown in Table 1, by adding the stabilizers, viability and heat stability of the samples were improved by the concentration at levels of 2 and 3 but reduced at level 4 for all of the stabilizers, except for lactose that it was seen uptrend even at level 4 and might be continued. According to the S/N ratio of the experiments and ranking of the factors (Table 2), the most effective stabilizer was lactose, Na-L-glutamate, and dextran, respectively. Moreover, the formulation of BCG bulk was re-finalized and then all three stabilizers, including lactose, Na-L-glutamate, and dextran were chosen at the concentration of 1%. The finalized BCG vaccine was tested again for viability, TS, reconstitution time, and skin reaction in guinea pigs. No significant difference (*P*≤0.05) was found in comparison to the BCG vaccine of experiment 10 in viability and TS, while meeting the WHO requirements and the pharmacopoeia (data not shown).

**Table 2:** Taguchi analysis for CFU versus G, L, T, D, N, and the main effect for signal-to-noise ratio and mean

Level 1	Signal-to-noise ratio					Mean				
	
	G	L	T	D	N	G	L	T	D	N
1	25.76	29.13	27.70	27.62	28.19	21.50	34.25	29.17	28.33	31.33
2	31.50	30.04	29.76	30.09	29.24	37.75	35.00	32.92	33.67	31.50
3	33.88	30.55	30.57	30.96	31.25	49.83	34.83	35.33	37.92	37.00
4	28.19	29.60	31.30	30.65	30.64	26.50	31.50	38.17	35.67	35.75
Delta	8.11	1.42	3.59	3.34	3.06	28.33	3.50	9.00	9.58	5.67
Rank	1	5	2	3	4	1	5	3	2	4

G, glucose; L, lactose; T, trehalose; D, dextrin; N, monosodium glutamate

## DISCUSSION

It has been estimated that less than 80% of biomass of BCG bacilli may be damaged or killed during homogenization and freeze-drying process in BCG vaccine production that affects the viability and heat stability of BCG vaccine. The aim of this study was the elevation of TS of Iranian freeze-dried BCG vaccine by improving the formulation of BCG final bulk prior to freeze drying.

Heat resistance or TS of BCG vaccine with survival rate usually over 20% is important as it shows uniformity and consistency of production and also stability of vaccine for transportation to tropical area[7,9]. BCG was the first vaccine that WHO established a requirement for its thermal stability (TS)[6,9]. TS is known to be dependent on the strain type, formulation of bulk, and freeze-drying process. BCG strains differ in their *in vitro* characteristics such as growth, morphology, heat stability and viability tested by CFU, and the ATP content[7,9]. French and Danish BCG strains gave a similar viability after freeze-drying[7]. TS of the vaccines prepared from various BCG strains was measured to select the heat-stable strains. The obtained results showed that although Danish 1331 and Pasteur 1173p2 were the strongest in immunogenicity, Japanese strain gave the highest TS at 37°C during 28 days. Japanese strain is the most heat-resistant strain with more than 39% of the viable units after heat exposure, followed by French (29% survival), Danish (27%), and Glaxo (11%) strains[6,7,9].

While all of the BCG strains are originally similar, it seems that among the main factors including BCG strains and lyophilization and stabilization techniques, the latter is probably more effective on TS[6].

In a study by Lugosi[9] on the heat stability of 13 kinds of BCG vaccines, the viability unit value of l0^6^/ml of the sample vaccines at day 0 was decreased in parallel but at day 28, only six vaccines showed a survival rate over 20%.

To enhance the heat-stability of the freeze-dried BCG vaccine, a number of stabilizers were added to the final bulk of the vaccine. Glucose and dextran had a good effect on TS of BCG vaccine in Copenhagen and French strain vaccines[14]. However, among these stabilizers, sodium glutamate had the highest effectiveness on the TS of the vaccine[4,15]. Concentration of the carrier is significantly influential for the survival of bacilli during freeze-drying. Vaccine containing 1% of sodium glutamate was characterized by best thermostability, homogeneity, and the high survival of bacilli during freeze-drying[7,9,11,17].

To solve the problem of low TS of Danish BCG vaccine, Ungar *et al*.[18], in preparation of the vaccine, resuspended harvested cells in a medium consisting of an 8.3% aqueous solution of dextran with 7.5% glucose prior to freeze-drying[18]. They did not report reactogenicity of the sample vaccines.

Complete survival of BCG vaccine was obtained by Gheorghiu *et al*.[8] when the BCG bacilli in glycerol solution were frozen and storage at -70°C for many years. Although freeze-drying kills more than 50% of live bacilli in the fresh suspension[22], storage of vaccine in -70ºC is not applicable for manufacturers and vaccinators.

The effect of 62 preservatives on the stability of BCG vaccine was investigated by Miller and Goodner[16]. The results indicated that the effectiveness of five carbohydrates (sucrose, lactose, glucose, galactose, and trehalose) and two organic acid salts (sodium glutamate and sodium aspartate) were more than other preservatives, which is in line with our results[16]. However, bacterial viability was lower in the presence of trehalose in comparison with other sugars, the same as the results obtained in the present study. Adding sugars at high concentration to final bulk increased viability during lyophilization but these concentrations were not applicable for BCG vaccine preparation[19]. Ungar and colleagues[18] reported that the heat stability of BCG vaccine were improved with adding glucose and dextran into vials during freeze-drying at the concentration of 5.7 and 3.8%, respectively[18]. Glutamate, as an amino acid, was added into the vials of all kinds of BCG vaccine as a protective excipient for freeze-drying. The results showed that glutamate improved the stability of BCG vaccine in a previous investigation[4]. BCG vaccine from Merck (USA) contains lactose and that from Thailand composes of sodium L-glutamate, dextran, dextrose, and trace amount of glycerin as stabilizer[8,20-22].

The results from this study indicated that combination of lactose, dextran, and glutamate at specified concentrations lower than those used in other studies improved the vaccine stability more than each one alone. The results also indicated that maximum S/N ratio equal to maximum viability and heat stability were obtained by adding lactose, dextran, and sodium glutamate while glucose and trehalose had not a significant effect on improvement of TS.

Freeze-drying is an effective method of preserving live-attenuated bacterial vaccines such as BCG vaccine in pharmaceutical industry. Some stabilizers or preservatives could be added to the final bulk to increase the TS and viability of Iranian BCG vaccine during freeze-drying.

## References

[1] Ritz N, Dutta B, Donath S, Casalaz D, Connell TG, Tebruegge M, Robins-Browne R, Hanekom WA, Britton WJ, Curtis N (2012). The influence of bacille Calmette-Guerin vaccine strain on the immune response against tuberculosis:a randomized trial. American journal of respiratory and critical care medicine.

[2] Elias D, Britton S, Aseffa A, Engers H, Akuffo H (2008). Poor immunogenicity of BCG in helminth infected population is associated with increased *in vitro* TGF-βproduction. Vaccine.

[3] Hubeau C, Singer M, Lagranderie M, Marchal G, Vargaftig B (2003). Extended freeze-dried Mycobacterium bovis Bacillus Calmette–Guérin induces the release of interleukin-12 but not tumour necrosis factor-α by alveolar macrophages, both *in vitro* and *in vivo*. Clinical and experimental allergy.

[4] Yamamoto S, Yamamoto T (2007). Historical review of BCG vaccine in Japan. Japanese journal of infectious diseases.

[5] World health organization (2006). Temperature sensitivity of vaccines.

[6] World health organization (1978). Requirements for dried BCG vaccine.

[7] Gheorghiu M, Lagrange PH (1983). Viability, heat stability and immunogenicity of four BCG vaccines prepared from four different BCG strains. Annals of immunology (Paris).

[8] Gheorghiu M, Lagranderie M, Balazuc AM (1996). Stabilization of BCG vaccine *Developments in biological standardization journal*.

[9] Lugosi L (1984). Multiple comparison of dried BCG vaccines:stability at 37 C and persistence of strains in the mouse spleen. Vaccine.

[10] Ito T (1954). The moisture content of dried BCG vaccine;relationship between moisture content and viable units. The science reports of the research institutes, Tohoku University Ser C. Medicine Tohoku Daigaku.

[11] Janaszek W (1990). Evaluation of thermostability of lyophilized BCG vaccines by using an accelerated thermal degradation test. Medycyna doswiadczalna i mikrobiologia.

[12] Q An Y (1991). Application and examination of bioluminescent technique in the quality identification of BCG vaccines. Chinese journal of tuberculosis and respiratory diseases.

[13] Janaszek W (1991). Investigation of factors determining stability of BCG vaccine. Medycyna doswiadczalna i mikrobiologia.

[14] Farmer P, Muggleton PW, Ungar J (1956). Freeze-dried B.C.G. vaccine;methods adopted in preparation of a standard product. British medical journal.

[15] Brandau DT, Jones LS, Wiethoff CM, Rexroad J, Middaugh CR (2003). Thermal stability of vaccines. Journal of pharmaceutical sciences.

[16] Miller R, Goodner K (1953). Studies on the stability of lyophilized BCG vaccine. The Yale journal of biology and medicine.

[17] Janaszek W (1992). Investigation of factors determining stability of BCG vaccine. Medycyna doswiadczalna i mikrobiologia.

[18] Ungar J, Farmer P, Muggleton PW (1956). Freeze-dried BCG vaccine. British medical journal.

[19] Argüelles JC (2000). Physiological roles of trehalose in bacteria and yeasts:a comparative analysis. Archives of microbiology.

[20] Cho C, Obayashi Y (1956). Effect of adjuvant on preservability of dried BCG vaccine at 37°C. Bulletin of the world health organization.

[21] (1957). WHO Tuberculosis Research Office and Research Institute of the Japan Anti-Tuberculosis Association. A field trial of freeze-dried glutamate BCG vaccine:Preliminary report. Bulletin of the world health organization.

[22] Obayashi Y, Cho C, Yoshioka T, Kawasaki J, Shimao T (1957). Effect of storage at 37°C on allergenic potency of dried BCG vaccine. Bulletin of the world health organization.

